# RETRACTED: Hussain et al. The Effect of 600 keV Ag Ion Irradiation on the Structural, Optical, and Photovoltaic Properties of MAPbBr_3_ Films for Perovksite Solar Cell Applications. *Materials* 2022, *15*, 5299

**DOI:** 10.3390/ma18071623

**Published:** 2025-04-02

**Authors:** Saddam Hussain, Norah Alwadai, Muhammad I. Khan, Muhammad Irfan, Ikram-ul-Haq, Hind Albalawi, Aljawhara H. Almuqrin, Maha M. Almoneef, Munawar Iqbal

**Affiliations:** 1 Department of Physics, University of Lahore, Lahore 53700, Pakistan; saddamhussain45270@gmail.com (S.H.); muhammad.irfan6252@gmail.com (M.I.); ikrmhaq@gmail.com (I.-u.-H.); 2 Department of Physics, College of Sciences, Princess Nourah bint Abdulrahman University, P.O. Box 84428, Riyadh 11671, Saudi Arabia; malbalawi@pnu.edu.sa (H.A.); ahalmoqren@pnu.edu.sa (A.H.A.); mmalmoneef@pnu.edu.sa (M.M.A.); 3 Department of Chemistry, University of Lahore, Lahore 53700, Pakistan

*Materials* retracts the article “The Effect of 600 keV Ag Ion Irradiation on the Structural, Op-tical, and Photovoltaic Properties of MAPbBr_3_ Films for Perovksite Solar Cell Applications” [1], cited above. 

Following publication, concerns were brought to the attention of the publisher regarding the validity of the XRD data presented in this article [1].

Adhering to our complaints procedure, an investigation was conducted by the Editorial Office and Editorial Board. While the authors fully cooperated with the Editorial Office during the investigation, they were unable to satisfactorily explain the presence of a number of significant anomalies within the raw XRD data provided. As a result, the Editorial Board and Editor-in-Chief were unable to confirm the reliability of the findings and subsequently decided to retract this paper as per MDPI’s retraction policy (https://www.mdpi.com/ethics#_bookmark30).

This retraction was approved by the Editor-in-Chief of the journal *Materials*.

The authors disagree with this retraction.
